# Pediatric psychotropic prescribing and polypharmacy, 2019-2022: a retrospective electronic health record study in Qatar

**DOI:** 10.3389/fpsyt.2026.1944247

**Published:** 2026-10-28

**Authors:** Dina Moustafa Abo El-Ella, Dhoha Dhieb, Yousef Kanan, Sazgar Hamad, Kholoud Bastaki

**Affiliations:** 1 Pharmaceutical Sciences Department, College of Pharmacy, QU Health, Qatar University, Doha, Qatar; 2 Department of Psychiatry, Hamad Medical Corporation, Doha, Qatar

**Keywords:** antidepressants, antipsychotics, pediatric psychopharmacology, polypharmacy, psychotropic medications

## Abstract

**Background:**

Real-world evidence on pediatric psychotropic prescribing and polypharmacy in Gulf health systems remains limited. Characterizing medication-class patterns and multi-class prescribing is essential for medication safety surveillance in children and adolescents. This study examined psychotropic prescribing, potential polypharmacy, and recorded medication combinations among children and adolescents using public-sector electronic health records in Qatar.

**Methods:**

A retrospective observational study used electronic health record data from Hamad Medical Corporation and the Primary Health Care Corporation, Qatar’s principal public healthcare providers, from January 2019 to December 2022. Children and adolescents aged 0–17 years with at least one psychotropic prescription were included. Medications were classified as antidepressants, antipsychotics, anxiolytics, mood stabilizers, or attention-deficit/hyperactivity disorder (ADHD) medications. Potential cross-class polypharmacy was assessed as the recording of two or more medication classes per patient during the study period; available duration information did not permit confirmation of concurrent exposure. Logistic regression examined patient-level associations with this outcome.

**Results:**

The dataset included 2,941 eligible prescription records corresponding to 2,221 unique patients. The proportion of prescriptions for ADHD medications decreased from 28.7% in 2019 to 13.7% in 2022, whereas the proportion assigned to anxiolytics increased from 26.0% to 37.5%. ADHD medications predominated among prescriptions for children aged 5–11 years, while antidepressants and antipsychotics predominated among those for adolescents aged 12–17 years. The potential-polypharmacy criterion was met by 322 patients (14.5%), reaching 21.6% among adolescents. In a gender-adjusted model restricted to patients aged 1–17 years, each additional year of age was associated with higher odds of meeting the criterion (adjusted OR 1.13, 95% CI 1.10-1.16). After excluding patients with prescription-linked seizure disorder or cerebral palsy, the corresponding proportion was 16.96% (259/1,527). Among patients meeting the criterion, antidepressant-antipsychotic and ADHD medication-antipsychotic pairs were most frequent; methylphenidate-risperidone was the most common recorded individual-drug pair.

**Conclusions:**

This study characterizes pediatric psychotropic prescribing and potential polypharmacy in Qatar’s public healthcare records and helps address the limited regional evidence from Gulf health systems. The observed patterns identify potential medication-safety surveillance priorities, particularly among adolescents and patients with antipsychotic-containing medication histories. Further assessment incorporating exposure duration and clinical outcomes is needed to establish concurrency and clinical significance.

## 1 Introduction

Mental and neurodevelopmental disorders are major contributors to morbidity in children and adolescents. Their onset and clinical presentation vary across development: attention-deficit/hyperactivity disorder, autism spectrum disorder, and related neurodevelopmental conditions are commonly identified in childhood, whereas depressive, anxiety, behavioral, and emerging severe mental disorders become more prominent during adolescence (1–3). These developmental differences are important when interpreting pediatric psychotropic prescribing, because medication use may reflect different indications and treatment pathways across age groups. Psychotropic medication prescribing in children and adolescents has increased in many countries during recent decades, although rates vary substantially by healthcare system, medication class, age group, diagnostic practice, and prescribing regulation (4–7). Prescription and dispensing data are commonly used in pharmacoepidemiology to monitor medication utilization and may provide indirect information about changing treatment patterns, service access, and population mental health needs (8). Psychotropic polypharmacy is an important additional dimension of pediatric psychopharmacology. Concurrent use of two or more psychotropic medication classes may reflect comorbidity, neurodevelopmental complexity, symptom severity, treatment augmentation, cross-titration, or specialist-led management (9–13). However, pediatric psychotropic polypharmacy also raises medication-safety concerns because evidence for many medication combinations remains limited, and cumulative adverse effects, drug-drug interactions, metabolic complications, neurological adverse effects, endocrine effects, sedation, and cardiovascular risks may be clinically important (9–13).

Medication-class and individual-drug analyses can therefore complement prescription counts when characterizing complexity. Although concurrent exposure is central to the clinical interpretation of polypharmacy, prescription-based identification of patients with multiple medication classes recorded can identify patterns warranting further assessment. In this study, these patterns were examined as indicators of potential cross-class polypharmacy because available exposure-duration information did not permit confirmation of concurrency. The class-level approach describes prescribing across broad therapeutic categories but does not capture patients receiving multiple medications exclusively within one class.

The COVID-19 pandemic disrupted child and adolescent mental health and healthcare delivery. Increased psychological distress, reduced access to routine services, changes in consultation practices, and expansion of telemedicine were reported internationally (14–17). Studies also described changes in medication utilization during the pandemic, including pediatric psychotropic prescribing (18–20). These developments provide context for the 2019–2022 observation period. However, the present study was not designed to establish a causal effect of the pandemic on prescribing. Evidence on pediatric psychotropic prescribing and polypharmacy remains limited in the Middle East and Gulf region. This gap is important because prescribing patterns may be influenced by population demographics, healthcare organization, referral pathways, availability of specialist child and adolescent mental health services, and access to non-pharmacological care. Qatar provides an informative setting for examining these questions. Hamad Medical Corporation and Primary Health Care Corporation are the principal public healthcare providers and use an integrated electronic health record system that captures prescribing across a large public-sector population. Local studies have described changes in child and adolescent mental health service activity during the pandemic, broader mental health and psychotropic medication trends in Qatar, ADHD pharmacotherapy in local child and adolescent mental health services, and national telepsychiatry and helpline initiatives (21–25). A previous Qatar-based electronic health record study examined broad mental health and psychotropic medication trends in the general population during the COVID-19 pandemic (22). The present study differs by focusing specifically on children and adolescents aged 0–17 years and examining patient-level medication-class patterns and recorded medication pairs alongside prescription-level utilization.

The objective of this study was to characterize pediatric psychotropic prescribing and prescription-based indicators of potential cross-class polypharmacy in Qatar between January 2019 and December 2022. We assessed temporal prescribing patterns, differences by age and gender, prescription-linked diagnoses, first observed medication class, and recorded medication combinations. These analyses were intended to provide descriptive and associational evidence relevant to pediatric medication-safety surveillance in a Gulf public healthcare system.

## 2 Methods

### 2.1 Study design and population

This retrospective observational study used electronic health record data from Hamad Medical Corporation and the Primary Health Care Corporation, the two principal public healthcare providers in Qatar. Together, these institutions serve approximately 90% of the national population (22). Eligible medication records were extracted from the Cerner electronic health record system for prescriptions recorded through public pharmacy services between January 2019 and December 2022.

Eligible patients were Qatari and non-Qatari children and adolescents aged 0–17 years who received at least one psychotropic medication prescription during the observation period. The prescribing dataset contained 2,941 eligible prescription records corresponding to 2,221 unique pediatric patients identified using the anonymized PERSON_ID variable. Among these patients, 465 contributed more than one prescription record, accounting for 720 records beyond the first record per patient. Eligible repeat prescriptions were retained for prescription-level summaries, while patient-level analyses used one analytical record per unique patient. For prescription-level analyses, age was calculated at the time of prescription and grouped as younger than 1 year, 1–4 years, 5–11 years, and 12–17 years. For patient-level analyses, age was defined at the first observed eligible prescription during the study period.

### 2.2 Medication and diagnosis classification

Psychotropic medications were identified from pharmacy records and assigned to five study-defined categories: antipsychotics, anxiolytics, antidepressants, mood stabilizers, and ADHD medications. The mood-stabilizer category included lithium and selected antiepileptic agents also used as mood stabilizers. The medication-to-class mapping and corresponding Anatomical Therapeutic Chemical (ATC) codes are provided in **Supplementary Table S1**. Each medication was assigned to one analytical category for class-level counting. These categories describe the study classification and should not be interpreted as confirmation of treatment indication. Extracted variables included medication details, prescription date, age, gender, prescription-linked diagnosis and prescriber specialty or role. Diagnoses were extracted from electronic order-diagnosis fields and grouped into categories including seizure disorder, ADHD, autism spectrum disorder, depressive disorders, anxiety disorders, behavioral disorders, sleep-related disorders, bipolar disorder, psychotic disorders and unspecified psychiatric conditions. The diagnosis categories displayed in the temporal summaries are shown in **Supplementary Figure S1**. When multiple diagnoses were recorded, all relevant categories were retained. The diagnosis analysis was restricted to diagnoses linked to eligible prescription records. It therefore describes prescription-associated diagnostic context rather than complete patient comorbidity or confirmed medication indications. This restriction concerned the diagnosis analysis and did not exclude patients from the prescribing cohort.

### 2.3 Outcomes

The primary outcomes were psychotropic prescribing patterns and prescription-based indicators of potential cross-class psychotropic polypharmacy. Prescribing patterns were assessed using annual prescription proportions and monthly prescription counts by medication class. Annual class proportions were calculated using the total number of eligible prescription records in the corresponding year as the denominator. Potential cross-class psychotropic polypharmacy was assessed using a prescription-based measure, defined as the recording of medications from two or more distinct study classes for the same patient during 2019-2022. This measure identifies multi-class prescribing patterns relevant to polypharmacy surveillance but does not establish simultaneous medication use. The available extract contained dispensing dates and quantities but lacked sufficient information to reconstruct continuous exposure duration. Consequently, a 30- or 60-day overlap threshold could not be applied, and sequential prescribing, switching, and concurrent treatment could not be reliably distinguished.

Secondary outcomes included medication-class distribution by age group, gender, prescription-linked diagnosis, and first observed medication class. The first observed medication class was defined as the earliest psychotropic medication class recorded during the study period. It was not assumed to represent treatment initiation because prescribing before the study period was unavailable. Medication-class and individual-drug pairs were examined among patients meeting the potential-polypharmacy criterion. Patients with three or more recorded medication classes could contribute multiple class pairs; therefore, combination percentages were not mutually exclusive. Individual-drug pairs could include medications from the same class, but this subgroup analysis did not estimate same-class prescribing in the entire cohort. Recorded pairs did not necessarily indicate concurrent exposure.

### 2.4 Statistical analysis

All analyses were performed using R software, version 4.2.1. A P value <0.05 was considered statistically significant. Descriptive analyses summarized patient characteristics, prescription-linked diagnoses, psychotropic medication classes, dispensed prescriptions, and prescribing patterns by demographic characteristics and calendar year. Categorical variables were reported as frequencies and percentages.

Annual and monthly prescription counts, medication-class proportions, and prescription-linked diagnoses were summarized descriptively at the prescription-record level. Patient-level analyses used one analytical record per unique patient and included comparisons of first-observed medication-class distributions, medication-class counts, Spearman correlation, and logistic regression. Prescription-level summaries were not treated as independent-patient inferential analyses. Monthly prescription counts were examined using seasonal-trend decomposition with LOESS (STL). LOESS smoothing was also used to visualize prescribing trajectories. These methods were used for descriptive visualization rather than formal assessment of pandemic-related effects.

Patient-level associations between categorical variables were examined using χ² tests of independence, with Cramér’s V used to describe effect size. Comparisons of medication-class distributions by age and gender used the first observed medication class for each patient. Spearman’s rank correlation assessed the association between age at the first observed prescription and the number of distinct medication classes recorded during the study period.

The proportion meeting the potential-polypharmacy criterion was calculated using the 2,221 unique patients as the denominator. Patient-level logistic regression examined associations between this binary outcome, age, and gender. The outcome was coded as 1 for patients meeting the criterion and 0 otherwise. The categorical-age model used children aged 5–11 years and males as the reference categories. A separate model treated age continuously and adjusted for gender. Infants were retained in descriptive summaries but excluded from the reported age-and-gender regression models because no outcome events were observed in that group.

Collinearity and an exploratory age-group- by-gender interaction were examined using generalized variance inflation factors and a likelihood-ratio test, respectively. Odds ratios and 95% confidence intervals were reported, with adjusted and unadjusted estimates distinguished. The apparent area under the receiver operating characteristic curve (AUC), with its 95% confidence interval calculated using the DeLong method, was reported as an in-sample descriptive measure. The models were intended to examine associations rather than develop a clinical prediction tool.

A neurological-diagnosis sensitivity analysis excluded patients with any prescription-linked diagnosis categorized as seizure disorder or cerebral palsy during 2019-2022. The proportion meeting the potential-polypharmacy criterion and recorded medication-pair patterns were reassessed in the remaining patients. An additional sensitivity analysis was restricted to patients whose first observed prescription occurred on or after 1 July 2019. This date-based restriction did not establish continuous prior observation or incident treatment use.

Medication-pair frequencies were summarized among the 322 unique patients meeting the potential-polypharmacy criterion, using this subgroup as the denominator for combination percentages.

### 2.5 Ethical considerations

Data were anonymized before analysis, and direct patient identifiers were removed. Data handling followed institutional standards for confidentiality and secure data management. The study was approved by the institutional review boards of Hamad Medical Corporation (MRC-01-22-088) and the Primary Health Care Corporation (PHCC/DCR/2021/06/045). Individual informed consent was not required because the study used anonymized routinely collected electronic health record data.

## 3 Results

### 3.1 Study cohort and prescription-linked diagnoses

The study included 2,941 eligible psychotropic prescription records corresponding to 2,221 unique children and adolescents aged 0–17 years (**Figure 1**). Among these patients, 465 contributed more than one prescription record, accounting for 720 records beyond the first record per patient.

**Figure 1:**
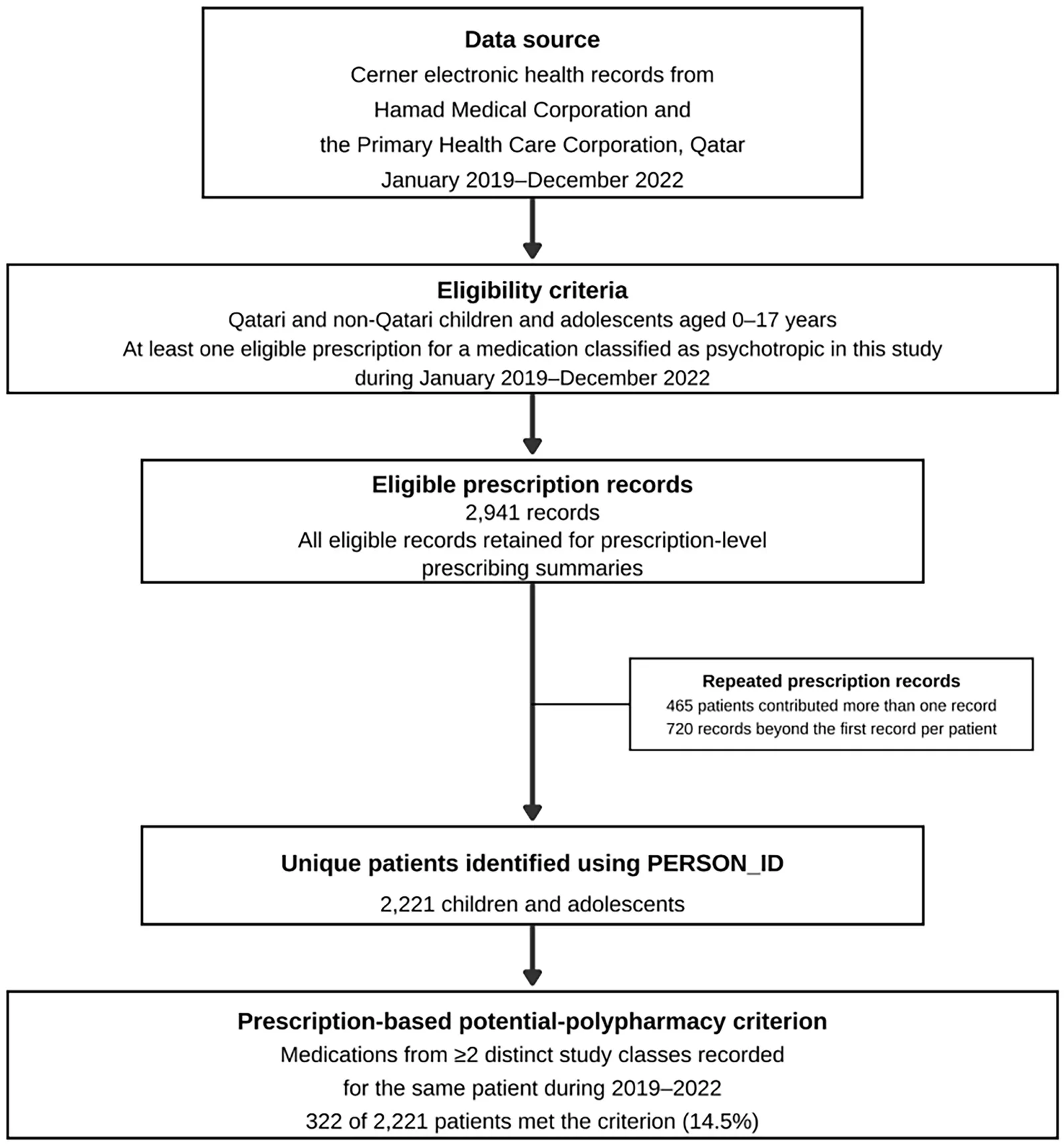
Construction of the analytical cohort, 2019-2022. The dataset comprised 2,941 prescription records from 2,221 unique patients; the 720 additional records represented repeat prescriptions, not patient exclusions. The potential-polypharmacy criterion was met by 322 patients (14.5%).

Prescription-linked diagnoses included psychiatric, neurodevelopmental and neurological conditions. Seizure disorder and ADHD were among the most frequently recorded diagnosis categories. Seizure disorder-linked prescriptions were concentrated in the study-defined anxiolytic and mood-stabilizer categories. Other commonly recorded categories included unspecified psychiatric conditions, autism spectrum disorder, depressive disorders, and anxiety disorders. Bipolar and psychotic disorders each represented less than 1% of prescription-linked diagnoses. Temporal patterns in the recorded diagnosis categories are presented in **Supplementary Figure S1**.

### 3.2 Temporal changes in psychotropic medication classes

The proportional distribution of recorded medication classes varied across calendar years (**Figure 2A**). ADHD medications accounted for 28.7% of recorded psychotropic prescriptions in 2019 and 13.7% in 2022, whereas the study-defined anxiolytic category accounted for 26.0% and 37.5%, respectively. The antidepressant proportion peaked at 26.5% in 2021. These percentages describe prescribing composition rather than patient-level treatment prevalence.

**Figure 2:**
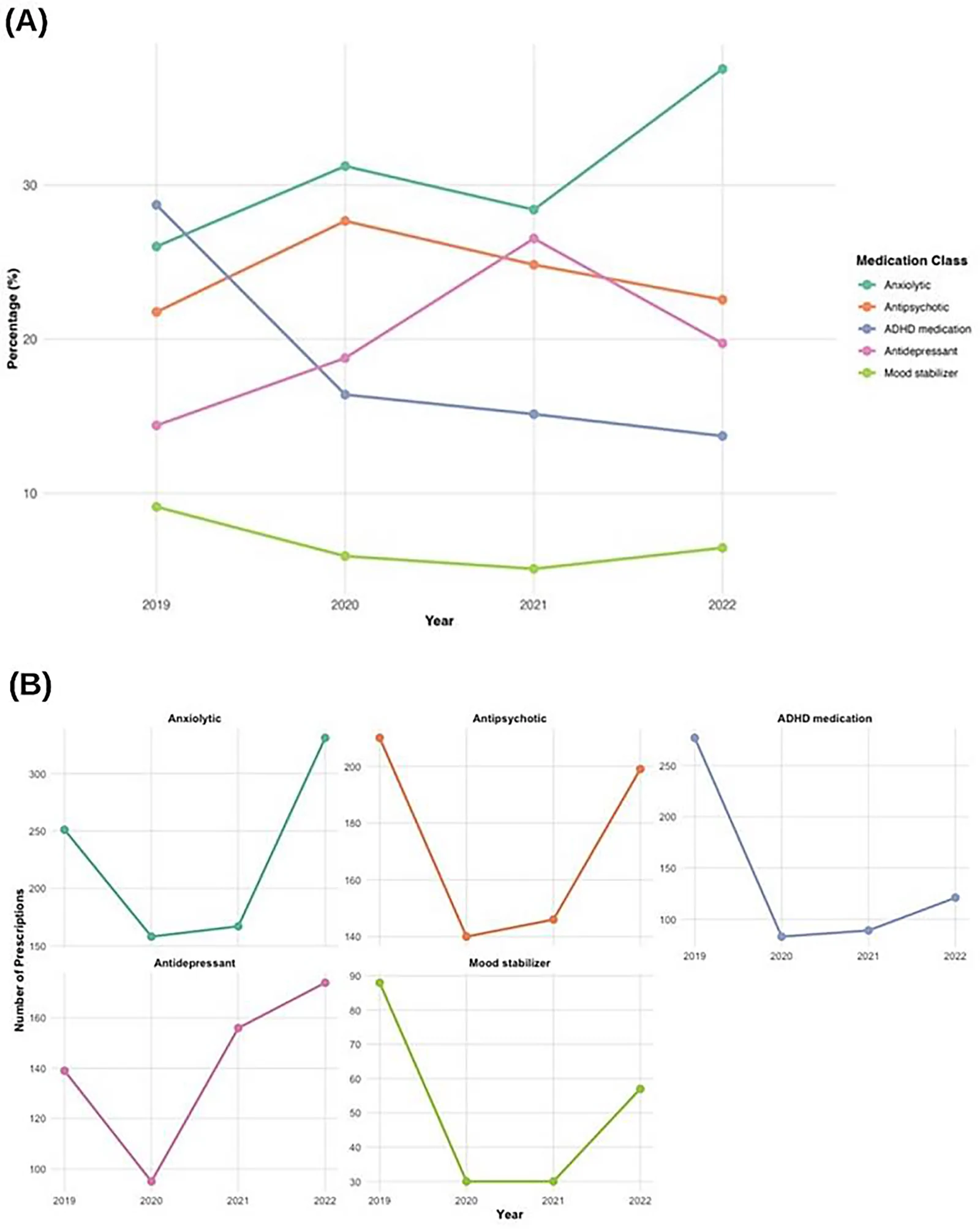
Annual psychotropic prescription proportions and counts, 2019-2022. **(A)** Proportion of eligible prescription records assigned to each medication category within each calendar year. **(B)** Absolute annual prescription-record counts by category. Percentages in panel A use all eligible prescription records in the corresponding year as the denominator. Patients could contribute multiple records. Both panels are descriptive.

Absolute annual prescription counts were lower in 2020 than in 2019 for most medication classes, followed by class-specific changes during 2021-2022 (**Figure 2B**). ADHD medication and mood-stabilizer counts remained below their 2019 levels in 2022, whereas anxiolytic counts increased by 2022 and antidepressant counts increased after 2020.

Monthly prescription counts and descriptive smoothed trends are presented in **Figure 3**. The smoothed trajectories showed lower prescribing counts during 2020, followed by class-specific patterns. ADHD medication and mood-stabilizer trends remained below their initial levels, while anxiolytics showed a later increase. The antidepressant smoothed trend reached its lowest point around early 2021 before increasing through 2022.

**Figure 3:**
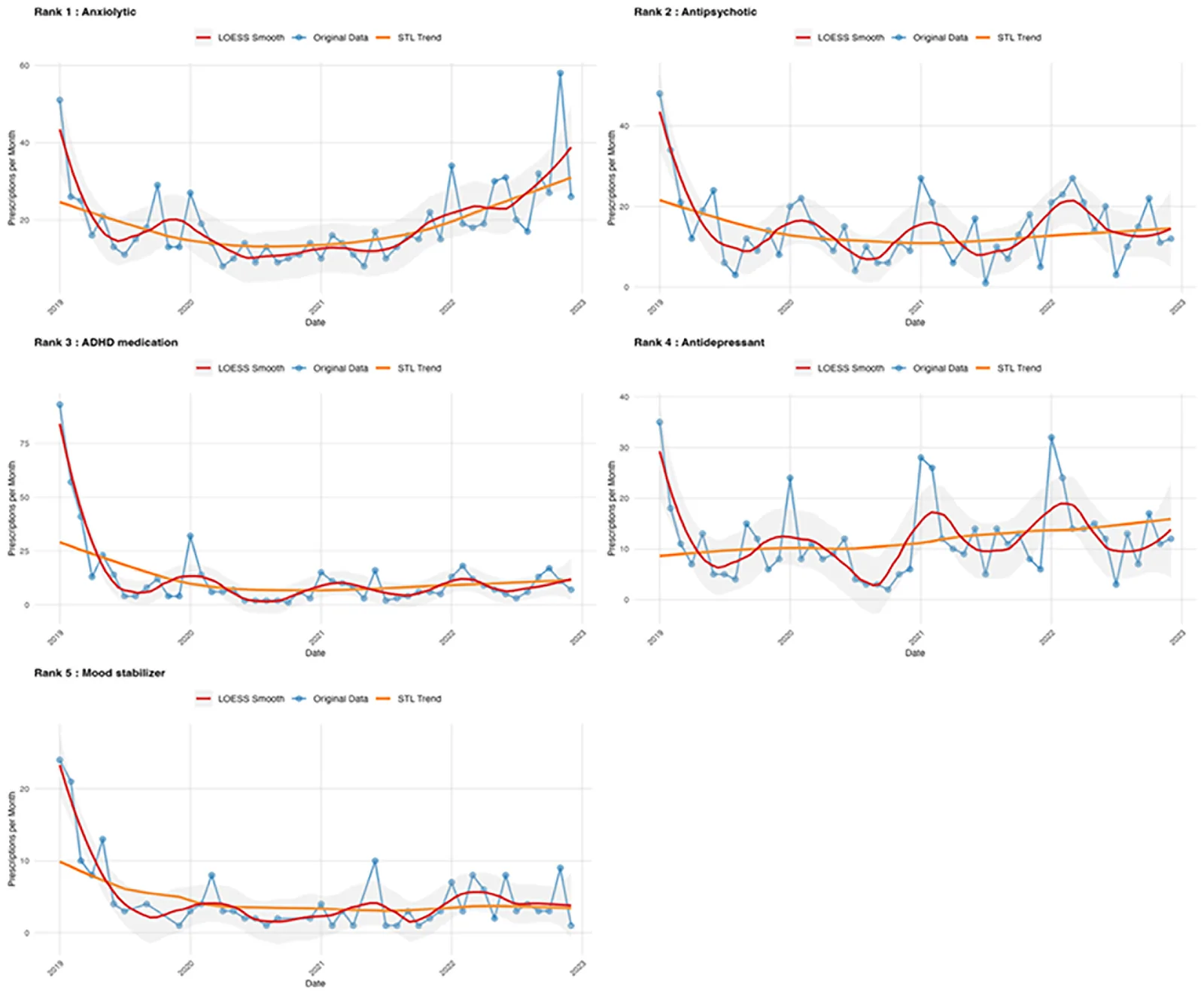
Monthly psychotropic prescription counts and descriptive trends, 2019-2022. Blue lines represent observed monthly prescription-record counts, orange lines the trend component from seasonal-trend decomposition using LOESS (STL), and red lines the LOESS smoother. Panels represent the five study medication categories. The curves describe temporal patterns and do not estimate causal pandemic effects.

### 3.3 Medication classes by prescription-linked diagnosis

ADHD medications accounted for 89.2% of ADHD-linked prescription records. Antidepressants accounted for 86.2% of depression-linked records and 77.5% of anxiety disorder-linked records; anxiolytics accounted for a further 9.9% of anxiety disorder-linked records.

Antipsychotics accounted for 75.7% of autism spectrum disorder-linked prescriptions and 70.7% of behavioral disorder-linked prescriptions. Among seizure disorder-linked prescriptions, 79.7% were assigned to the anxiolytic category and 18.6% to the mood-stabilizer category.

### 3.4 Age- and gender-specific prescribing patterns

Prescription-level medication-class distributions varied by age group (**Figure 4A**). In infants younger than 1 year, all eligible prescriptions were assigned to the study-defined anxiolytic category. This category also predominated among children aged 1–4 years, accounting for 93.3% of prescriptions. Among children aged 5–11 years, ADHD medications were most common (40.4%), followed by anxiolytics (28.9%) and antipsychotics (19.8%). Among adolescents aged 12–17 years, antidepressants and antipsychotics accounted for 33.9% and 32.4% of prescriptions, respectively. ADHD medications accounted for 14.6%, and anxiolytics for 11.5%.

**Figure 4:**
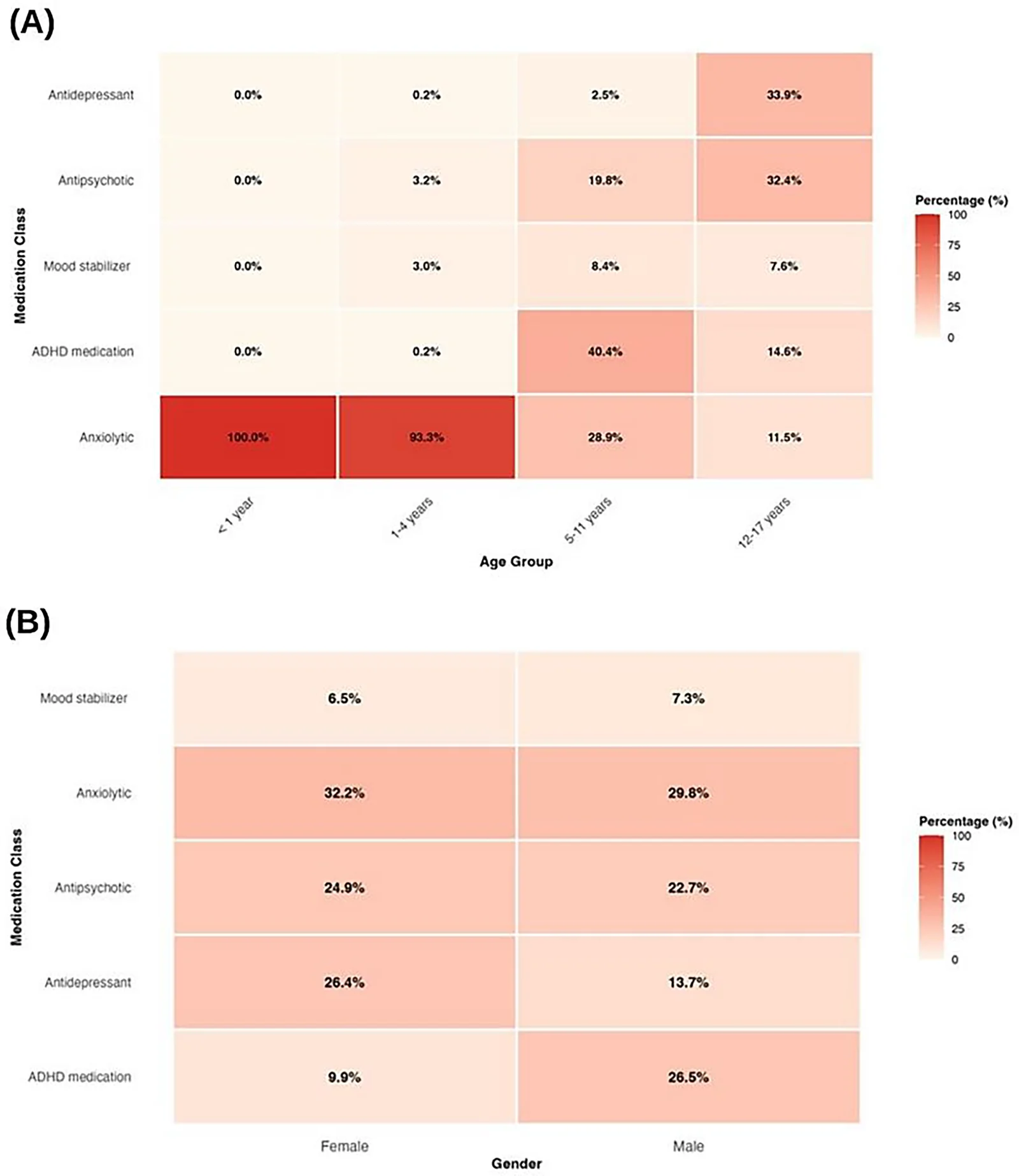
Prescription-level distribution of study medication categories by **(A)** age group and **(B)** gender, 2019-2022. Percentages are calculated within each age group or gender, using all eligible prescription records in that group as the denominator.

Prescription-level proportions also varied by gender (**Figure 4B**). ADHD medications accounted for a larger proportion of prescriptions among males than females (26.5% versus 9.9%), whereas antidepressants accounted for a larger proportion among females than males (26.4% versus 13.7%). Differences were smaller for the other medication categories. In separate patient-level comparisons using one first-observed medication class per patient, class distribution was associated with age group (χ² = 1,359.71, df = 12, P < 0.001; Cramér’s V = 0.452) and gender (χ² = 157.62, df = 4, P < 0.001; Cramér’s V = 0.266). Age was positively associated with the number of distinct medication classes recorded during the study period (Spearman’s ρ = 0.192, P < 0.001; n = 2,221; **Supplementary Figure S2**).

### 3.5 Frequency and correlates of potential psychotropic polypharmacy

Among 2,221 unique patients, 322 met the prescription-based criterion for potential cross-class polypharmacy, corresponding to 14.5%. The proportion meeting this criterion differed by age group (χ² = 109.72, df = 3, P < 0.001). No infants younger than 1 year met the criterion; the corresponding proportions were 1.99% among children aged 1–4 years, 12.39% among children aged 5–11 years, and 21.62% among adolescents aged 12–17 years (**Figure 5A**).

**Figure 5:**
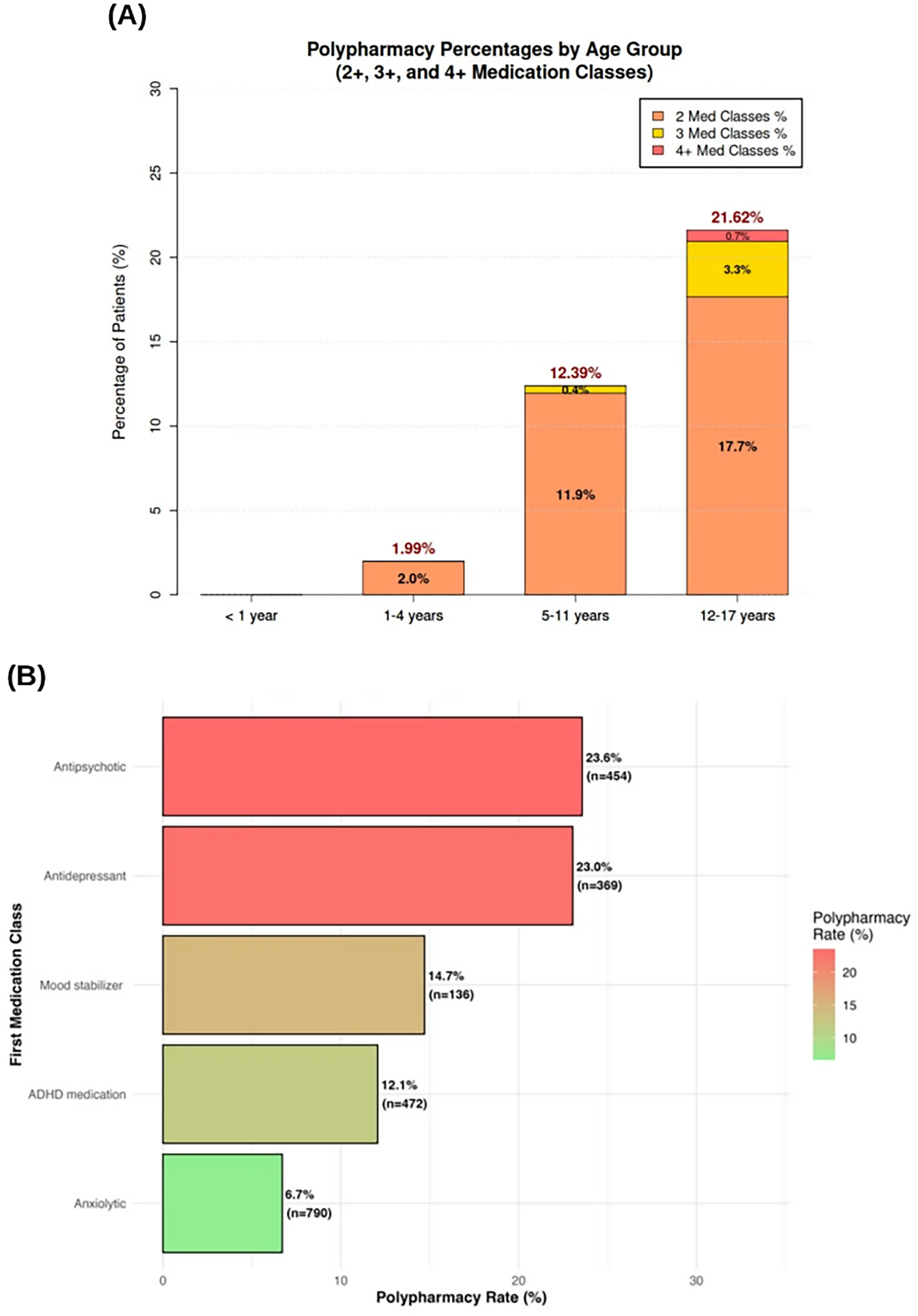
Potential cross-class polypharmacy by age group and first observed medication class. **(A)** Percentage of patients meeting the prescription-based criterion within each age group. **(B)** Percentage meeting the criterion within each first-observed medication-class group. First observed class does not necessarily represent treatment initiation. The criterion identifies multi-class prescribing during the observation period and does not establish concurrent exposure.

In the age-group model adjusted for gender, children aged 1–4 years had lower odds of meeting the criterion than children aged 5–11 years (adjusted OR 0.14, 95% CI 0.07-0.30), while adolescents aged 12–17 years had higher odds (adjusted OR 1.90, 95% CI 1.44-2.50). Infants were retained in descriptive summaries but excluded from the reported regression models because no events were observed in this group. In the separate gender-adjusted continuous-age model, each additional year of age was associated with higher odds of meeting the criterion (adjusted OR 1.13, 95% CI 1.10-1.16; P < 0.001).

The unadjusted proportion was higher among females than males (16.33% versus 13.16%; **Supplementary Figure S3**). The unadjusted female-versus-male OR was 1.29 (95% CI 1.02-1.63; P = 0.042). After adjustment for age group, the association was no longer statistically significant (adjusted OR 1.13, 95% CI 0.89-1.45; P = 0.317).

Collinearity was minimal, with adjusted generalized variance inflation factors of 1.01 for age group and 1.02 for gender. The exploratory age-group-by-gender interaction was not statistically significant (likelihood-ratio χ² = 0.61, df = 2, P = 0.737) and was not included in the final model. The model converged successfully. Its apparent AUC was 0.659 (95% CI 0.632-0.687; DeLong method), representing in-sample discrimination rather than validated predictive performance.

First observed medication class was associated with meeting the potential-polypharmacy criterion (χ² = 92.73, df = 4, P < 0.001; Cramér’s V = 0.204). The proportions were highest among patients whose first observed class was an antipsychotic or antidepressant (23.6% and 23.0%, respectively), followed by mood stabilizers (14.7%), ADHD medications (12.1%), and anxiolytics (6.7%; **Figure 5B**). These findings describe exploratory associations with first-recorded prescribing rather than treatment initiation.

In the neurological-diagnosis sensitivity analysis, 694 patients with a prescription-linked diagnosis of seizure disorder or cerebral palsy were excluded, leaving 1,527 patients. Of these, 259 met the potential-polypharmacy criterion (16.96%), compared with 322 of 2,221 patients (14.5%) in the full cohort. The principal recorded pairs were unchanged: antidepressant-antipsychotic and ADHD medication-antipsychotic remained the most frequent class-level pairs, and methylphenidate-risperidone remained the most frequent individual-drug pair.

In the sensitivity analysis restricted to patients first recorded on or after 1 July 2019, 211 of 1,615 patients met the criterion (13.07%). The highest proportions were observed among those whose first recorded class was an antipsychotic (76/335, 22.69%) or antidepressant (65/303, 21.45%). This date-based restriction did not establish incident treatment use.

### 3.6 Recorded medication pairs among patients meeting the potential-polypharmacy criterion

Among the 322 patients meeting the criterion, the most frequent recorded class-level pair was antidepressant-antipsychotic, observed in 140 patients (43.5%), followed by ADHD medication-antipsychotic in 75 patients (23.3%). Other frequently recorded pairs were anxiolytic-mood stabilizer (54 patients, 16.8%), antidepressant-anxiolytic (40 patients, 12.4%), and antipsychotic-anxiolytic (39 patients, 12.1%; **Figure 6A**). Patients with three or more recorded classes could contribute multiple pairs; therefore, percentages were not mutually exclusive. At the individual-drug level, methylphenidate-risperidone was the most frequent recorded pair (41 patients, 12.7%). Other common pairs included fluoxetine-olanzapine (9.0%), fluoxetine-risperidone (8.7%), aripiprazole-risperidone (8.7%), risperidone-sertraline (7.5%), atomoxetine-risperidone (7.1%), and aripiprazole-fluoxetine (7.1%; **Figure 6B**). Clobazam-valproic acid was recorded in 18 patients (5.6%). Individual-drug pairs were described within the subgroup meeting the cross-class criterion and could include same-class pairs. These findings do not estimate same-class prescribing in the entire cohort. Recorded pairs do not necessarily indicate concurrent exposure.

**Figure 6:**
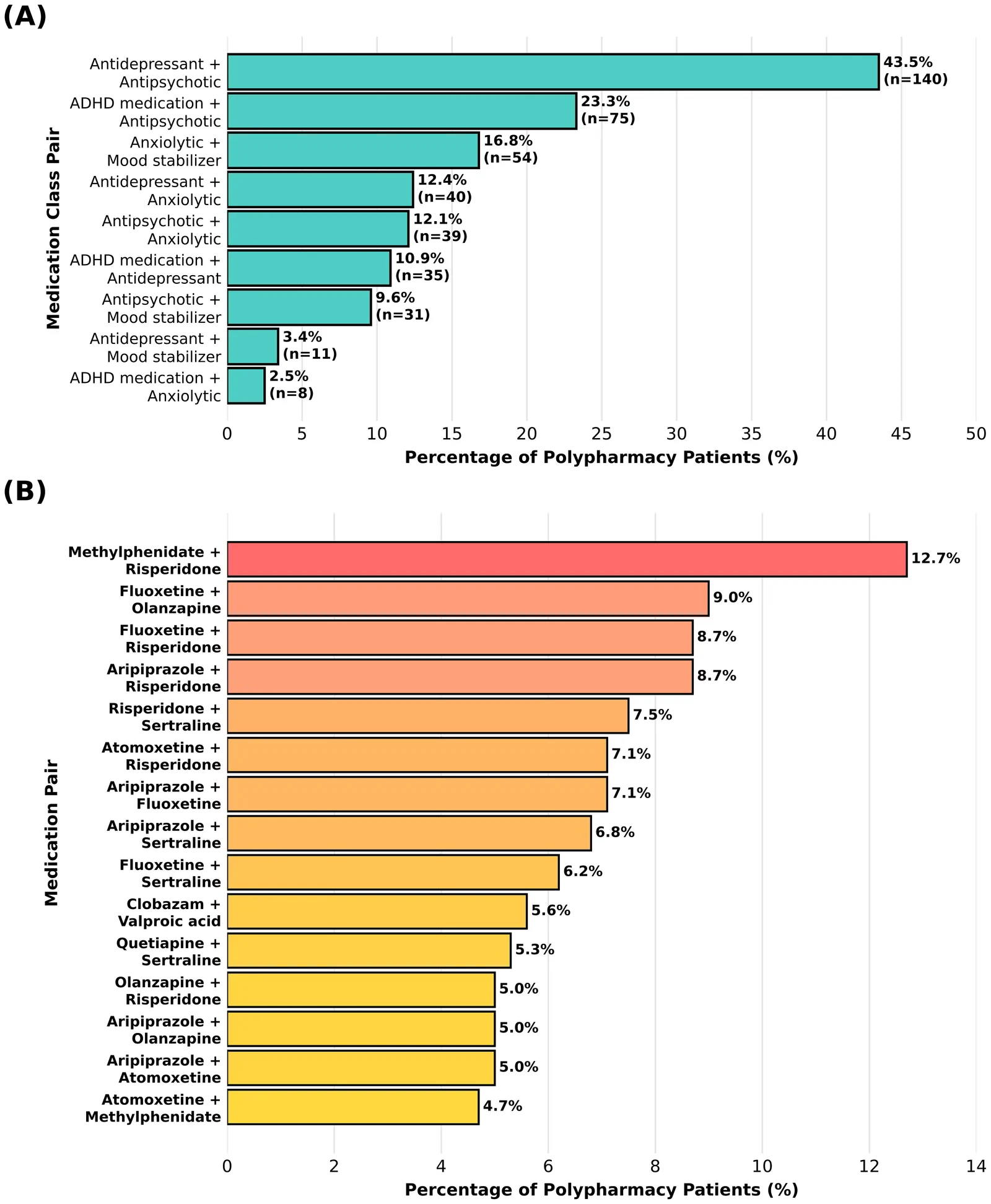
Recorded medication pairs among 322 patients meeting the potential-polypharmacy criterion. **(A)** Medication-class pairs. **(B)** Individual-drug pairs. Percentages use the 322-patient subgroup as the denominator and are not mutually exclusive.

## 4 Discussion

This public-sector EHR study provides real-world evidence on pediatric psychotropic prescribing and potential polypharmacy in Qatar, a setting where such data remain limited. Its principal contribution is the characterization of medication-class patterns and recorded medication pairs relevant to medication-safety surveillance. Between 2019 and 2022, the relative distribution of prescription records shifted across medication classes, including a lower proportion of ADHD medication prescriptions and a higher proportion assigned to anxiolytics in 2022 than in 2019; the antidepressant proportion peaked in 2021. Potential polypharmacy was more frequently observed among adolescents and patients whose first observed medication class was an antipsychotic or antidepressant. Antipsychotic-containing medication pairs were prominent. These findings identify potential priorities for surveillance and further clinical assessment rather than evidence of inappropriate prescribing or medication-related harm.

A key strength of this analysis is that it moves beyond prescription counts to describe patient-level medication-class patterns and recorded drug pairs. The prescription-based criterion identifies patients with multiple medication classes recorded during the observation period but does not establish concurrent exposure. These medication histories may reflect treatment complexity, comorbidity, changes in therapy, or concurrent prescribing; the available extract could not reliably distinguish these explanations. The study nevertheless adds regional evidence by identifying the medication classes and recorded drug pairs contributing to these patterns in a Gulf public healthcare system.

Direct comparison with international estimates requires caution. Published pediatric studies differ in age range, medication categories, inclusion of same-class combinations, minimum overlap duration, and denominator population. Previous methodological literature has emphasized that these differences substantially affect the interpretation of pediatric polypharmacy estimates (9, 13). In particular, the present prescription-based measure of potential polypharmacy should not be interpreted as directly equivalent to estimates requiring confirmed concurrent treatment. These differences limit conclusions about whether prescribing complexity in Qatar is higher or lower than that reported elsewhere.

The observed reduction in ADHD medication prescribing is a clinically relevant finding. ADHD assessment and treatment commonly involve developmental evaluation, school-based observations, caregiver and teacher reports, and continuity of follow-up. A reduction in recorded prescribing may therefore reflect changes in identification, referral, assessment, follow-up, or service access rather than a reduction in ADHD burden. This interpretation is relevant in Qatar, where local evidence indicates that ADHD pharmacotherapy may occur in the context of broader neurodevelopmental complexity, including coexisting autism spectrum disorder (23). The prominence of recorded ADHD medication-antipsychotic pairs may also reflect complex developmental or behavioral presentations, although the indications and timing of individual treatments were not confirmed. The observed antidepressant prescribing pattern also warrants cautious interpretation. In adolescents, antidepressant prescribing may reflect increased recognition or treatment of depressive and anxiety disorders, increased help-seeking, changes in referral thresholds, or greater clinical acuity among referred patients. Local child and adolescent mental health service data from Qatar reported changes in referrals and clinical activity during the pandemic period (21). The antidepressant pattern in the present study is therefore clinically plausible but should not be interpreted as a direct measure of depression prevalence. Prescription data cannot distinguish changes in disease incidence from delayed presentation, increased diagnosis, changes in access, or changes in prescribing practices. The anxiolytic findings illustrate an important methodological consideration in pediatric psychotropic pharmacoepidemiology. In younger children, particularly those younger than 5 years, prescribing within the study-defined anxiolytic category should be interpreted in light of the seizure-related diagnoses represented in the dataset. Some prescriptions may therefore reflect neurological rather than primary psychiatric treatment. Similarly, medications classified as mood stabilizers may have been prescribed for seizure-related indications. Medication identity, age, and diagnostic context should consequently be considered together rather than interpreting medication-category labels as confirmation of psychiatric indication. The proportion meeting the potential-polypharmacy criterion was higher after excluding patients with recorded seizure disorder or cerebral palsy (16.96% versus 14.50%). This restricted analysis provides additional context for interpreting the overall estimate. However, it does not confirm psychiatric indication for every remaining prescription and may exclude patients with both neurological and psychiatric conditions. Residual indication misclassification therefore remains possible.

The observed age-related patterns are consistent with developmental differences in prescribing. ADHD medications predominated among prescription records for children aged 5–11 years, whereas antidepressants and antipsychotics were more prominent among adolescents aged 12–17 years. The higher frequency of potential polypharmacy among adolescents suggests possible priorities for developmentally appropriate medication review. For children aged 5–11 years, potential priorities include coordination among families, schools, pediatrics, primary care, and developmental-behavioral services. For adolescents, relevant considerations include review of complex medication histories, monitoring of antipsychotic treatment, assessment of self-harm and mood-related risk when clinically indicated, and continuity of care during transition to adult services. For adolescents with ADHD, coordination between pediatric and adult services may be particularly relevant when psychiatric comorbidity and multi-specialty prescribing complicate medication management. Recent regional evidence from adults with ADHD in the United Arab Emirates similarly showed frequent psychiatric comorbidity and diagnosis across several clinical specialties (26).

The medication-pair analysis provides additional clinically relevant descriptive information. Antidepressant–antipsychotic and ADHD medication-antipsychotic pairs were the dominant class-level patterns, and risperidone appeared repeatedly among common individual-drug pairs. These medication histories may reflect ADHD with aggression or disruptive behavior, autism-associated irritability, severe emotional dysregulation, complex mood or anxiety disorders, or augmentation strategies in difficult-to-treat presentations. Sequential treatment is another possible explanation because concurrency was not confirmed. When antipsychotic-containing combinations are used concurrently, the indication, target symptoms, expected benefit, intended duration, previous non-pharmacological strategies, and monitoring plan should be explicit in the clinical record.

The appearance of risperidone across multiple recorded pairs highlights antipsychotic monitoring as a potential surveillance priority. Antipsychotics in children and adolescents are associated with metabolic, cardiovascular, neurological, endocrine, and sedative adverse effects. NICE guidance recommends baseline and follow-up monitoring of relevant physical health measures and adverse effects, including weight, metabolic parameters, movement disorders, and cardiovascular assessment when indicated (27). For children with autism-associated behavioral challenges, antipsychotic treatment should be considered within a broader management plan and reviewed carefully (28). These recommendations provide a relevant clinical framework for reviewing patients with recorded antipsychotic exposure, particularly when concurrent treatment with other psychotropic medications is confirmed.

The recording of two antipsychotics in the same patient’s medication history requires particular caution in interpretation. Pairs such as aripiprazole–risperidone may reflect sequential treatment, short-term cross-titration, or concurrent therapy. The available extract could not reliably distinguish these scenarios. Temporary overlap during switching may be clinically appropriate, whereas prolonged dual antipsychotic treatment requires careful justification and monitoring. Future EHR-based studies should therefore examine exposure duration, dose trajectories, switching windows, and persistence of same-class combinations.

The findings suggest potential surveillance priorities for Qatar’s child and adolescent mental health services. Patients with multiple recorded psychotropic medication classes could be considered for structured medication review to establish their current treatment and clarify whether prescribing represents concurrent use or sequential therapy. Confirmed antipsychotic-containing combinations may warrant specialist review where clinically appropriate. With suitable exposure-duration data, EHR-based tools could support identification of prolonged combinations and incomplete monitoring. Pharmacist-supported review could assist with medication reconciliation, adherence assessment, adverse-effect screening, caregiver counseling, and coordination across psychiatry, pediatrics, neurology, primary care, and pharmacy. Benzodiazepine and other study-defined anxiolytic prescribing may warrant separate assessment because of the mixture of psychiatric and neurological indications. The appropriate review frequency, feasibility, and clinical effects of these approaches require evaluation; they were not assessed in the present study.

The service implications extend beyond prescribing. During periods of healthcare disruption, children and adolescents may experience delayed assessment, interrupted follow-up, and reduced access to non-pharmacological interventions. International studies have reported changes in pediatric psychotropic prescribing after the onset of the COVID-19 pandemic (18, 19). In Qatar, telepsychiatry services and the National Mental Health Helpline were implemented to support access to mental healthcare (24, 25). Linking such services to medication follow-up pathways is a potential service consideration, particularly for adolescents with complex medication histories. However, the present descriptive temporal analyses cannot establish that pandemic-related disruption caused the observed prescribing patterns.

Remote care should be integrated with face-to-face assessment according to clinical and developmental needs. International guidance emphasizes considerations including severity, privacy, safeguarding, accessibility, family involvement, and patient preference (29, 30). For medication management, remote follow-up should support assessment of response, adherence, adverse effects, suicidality when relevant, and completion of indicated physical health monitoring. These considerations are particularly relevant for adolescents with mood or anxiety disorders, self-harm risk, behavioral dysregulation, or complex comorbidity.

This study has several strengths. It used real-world EHR data from Qatar’s principal public healthcare providers and included patients across the pediatric age range. The analysis examined temporal prescribing patterns, age and gender, prescription-linked diagnoses, first observed medication class, prescription-based indicators of potential polypharmacy, and individual medication pairs. It therefore complements prescription-count summaries and adds evidence from a Gulf health system where pediatric psychotropic prescribing remains insufficiently characterized.

Several limitations should be acknowledged. The findings concern eligible prescriptions recorded in the participating public healthcare systems and may not capture private-sector prescribing or treatment received elsewhere. Prescription-linked diagnoses do not necessarily establish the indication for individual medications, particularly benzodiazepines and antiepileptic agents. Although the neurological-diagnosis sensitivity analysis addressed part of this concern, residual indication misclassification remains possible. The extract did not contain sufficient information to reconstruct continuous exposure or confirm concurrent use. The potential-polypharmacy measure may therefore include sequential treatment and switching, and the proportion meeting the criterion should not be interpreted as the prevalence of confirmed concurrent polypharmacy. Because only prescribing from 2019 onward was available, patients already receiving treatment before the study period could not be distinguished from new users, and the first observed medication class may not represent treatment initiation. Patients contributed different numbers of prescription records, and prescription-level summaries may therefore be weighted towards patients with frequent prescribing. The regression models included only age and gender, so residual confounding by diagnosis, severity, prescriber specialty and other unmeasured factors is likely. Missing or incompletely recorded diagnoses and prescription details could not be assessed. The study design does not permit causal inference, including the effect of the COVID-19 pandemic. Finally, the findings reflect public-sector care in Qatar and may not be generalizable to private-sector settings or other health systems.

Future research should link prescribing trajectories with clinical outcomes, adverse events, emergency presentations, treatment discontinuation, and healthcare utilization. Priority questions include whether recorded antipsychotic-containing medication pairs represent concurrent treatment and, if so, whether that treatment is short-term or persistent; whether recommended monitoring is completed; and whether confirmed combinations are associated with measurable benefit or harm. Further work should also examine access to psychological, behavioral, family-based, and school-based interventions among children and adolescents with complex medication histories.

## 5 Conclusion

This retrospective public-sector EHR study characterized pediatric psychotropic prescribing and potential polypharmacy in Qatar during 2019-2022. Prescribing patterns varied by age, gender, diagnostic context, and medication class. The prescription-based criterion for potential cross-class polypharmacy was met by 14.5% of the 2,221 unique patients and was more frequently met among adolescents. The corresponding proportion was 16.96% after excluding patients with prescription-linked seizure disorder or cerebral palsy. Antidepressant-antipsychotic and ADHD medication-antipsychotic pairs were the most frequently recorded class-level combinations. These findings identify potential medication-safety surveillance priorities, but do not establish concurrent exposure, inappropriate prescribing, or medication-related harm. Further studies incorporating exposure duration, verified indications, and clinical outcomes are needed to distinguish concurrent treatment from sequential prescribing and evaluate the potential benefits of medication-review interventions.

## Data Availability

The raw data supporting the conclusions of this article will be made available by the authors, without undue reservation.
