# Supplementary material for: Pediatric psychotropic prescribing and polypharmacy, 2019-2022: a retrospective electronic health record study in Qatar
Source: Front Psychiatry. 2026 Oct 28;17:1944247. doi: 10.3389/fpsyt.2026.1944247 (PMC13753185; doi:10.3389/fpsyt.2026.1944247)
Supplement: Supplementary file 1 [file SupplementaryFile1.docx]

Supplementary Material

# 1 Supplementary Data

N/A

# 2 Supplementary Figures and Tables

## 2.1 Supplementary Figures

**Supplementary Figure S1. Prescription-linked diagnosis categories, 2019-2022.** (A) Annual prescription-record counts by linked diagnosis category. (B) Monthly counts for the ten most frequent categories; y-axes are scaled independently. A prescription record could contribute to more than one diagnosis category. ADHD, attention-deficit/hyperactivity disorder.

**Supplementary Figure S2. Age and number of distinct psychotropic medication classes.** Age at the first observed prescription and the number of distinct study medication classes recorded during 2019-2022 (one point per patient; n = 2,221). The orange line shows the median class count at each age. Spearman’s ρ = 0.192, P < 0.001.

**Supplementary Figure S3. Potential polypharmacy by gender.** Percentage of patients meeting the prescription-based potential-polypharmacy criterion by gender (n = 2,221), stratified by the number of recorded medication classes. The unadjusted proportion was 16.33% in females and 13.16% in males (unadjusted OR 1.29, 95% CI 1.02-1.63); the association was not statistically significant after adjustment for age group (adjusted OR 1.13, 95% CI 0.89-1.45).

**Supplementary Tables**

**Supplementary Table S1.** Medication-to-category mapping and Anatomical Therapeutic Chemical (ATC) codes used to classify psychotropic prescriptions.

| **Study category** | **Medication** | **Pharmacological group** | **ATC code** |
| --- | --- | --- | --- |
| **Antipsychotics** | Amisulpride | Second-generation (atypical) antipsychotic | N05AL05 |
|  | Aripiprazole | Second-generation (atypical) antipsychotic | N05AX12 |
|  | Olanzapine | Second-generation (atypical) antipsychotic | N05AH03 |
|  | Paliperidone | Second-generation (atypical) antipsychotic | N05AX13 |
|  | Quetiapine | Second-generation (atypical) antipsychotic | N05AH04 |
|  | Risperidone | Second-generation (atypical) antipsychotic | N05AX08 |
|  | Chlorpromazine | First-generation (typical) antipsychotic | N05AA01 |
|  | Flupentixol | First-generation (typical) antipsychotic | N05AF01 |
|  | Fluphenazine | First-generation (typical) antipsychotic | N05AB02 |
|  | Haloperidol | First-generation (typical) antipsychotic | N05AD01 |
|  | Prochlorperazine | First-generation (typical) antipsychotic | N05AB04 |
|  | Trifluoperazine | First-generation (typical) antipsychotic | N05AB06 |
|  | Zuclopenthixol | First-generation (typical) antipsychotic | N05AF05 |
| **Anxiolytics** | Alprazolam | Benzodiazepine | N05BA12 |
|  | Clobazam | Benzodiazepine | N05BA09 |
|  | Clonazepam | Benzodiazepine | N03AE01 |
|  | Diazepam | Benzodiazepine | N05BA01 |
|  | Lorazepam | Benzodiazepine | N05BA06 |
|  | Midazolam | Benzodiazepine | N05CD08 |
|  | Buspirone | Azapirone (non-benzodiazepine anxiolytic) | N05BE01 |
|  | Hydroxyzine | Antihistamine | N05BB01 |
| **Antidepressants** | Escitalopram | SSRI | N06AB10 |
|  | Fluoxetine | SSRI | N06AB03 |
|  | Fluvoxamine | SSRI | N06AB08 |
|  | Paroxetine | SSRI | N06AB05 |
|  | Sertraline | SSRI | N06AB06 |
|  | Duloxetine | SNRI | N06AX21 |
|  | Venlafaxine | SNRI | N06AX16 |
|  | Amitriptyline | TCA | N06AA09 |
|  | Clomipramine | TCA | N06AA04 |
|  | Imipramine | TCA | N06AA02 |
|  | Mirtazapine | NaSSA | N06AX11 |
|  | Trazodone | SARI | N06AX05 |
| **Mood stabilizers** | Lithium | Lithium salt | N05AN01 |
|  | Carbamazepine | Antiepileptic | N03AF01 |
|  | Lamotrigine | Antiepileptic | N03AX09 |
|  | Valproic acid | Antiepileptic | N03AG01 |
| **ADHD medications** | Methylphenidate | Psychostimulant | N06BA04 |
|  | Atomoxetine | Selective noradrenaline reuptake inhibitor (non-stimulant) | N06BA09 |

**Note:** Study categories are analytical groupings and do not necessarily correspond to a single ATC therapeutic subgroup or establish treatment indication. Clonazepam (N03AE01) and midazolam (N05CD08) were assigned to the study-defined anxiolytic category, while lithium and selected antiepileptic agents were grouped as mood stabilizers.

SSRI, selective serotonin reuptake inhibitor; SNRI, serotonin-noradrenaline reuptake inhibitor; TCA, tricyclic antidepressant; NaSSA, noradrenergic and specific serotonergic antidepressant; SARI, serotonin antagonist and reuptake inhibitor.
